# 3-Hydroxybutyrate Ameliorates the Progression of Diabetic Nephropathy

**DOI:** 10.3390/antiox11020381

**Published:** 2022-02-14

**Authors:** Jeeyoun Jung, Woo Yeong Park, Yun Jin Kim, Mikyung Kim, Misun Choe, Kyubok Jin, Ji Hae Seo, Eunyoung Ha

**Affiliations:** 1 Clinical Medicine Division, Korea Institute of Oriental Medicine, Daejeon 34054, Korea; jjy0918@kiom.re.kr; 2 Department of Internal Medicine, Keimyung University School of Medicine, Daegu 42601, Korea; parkwy2015@dsmc.or.kr; 3 Department of Biochemistry, Keimyung University School of Medicine, Daegu 42601, Korea; salang0620@nate.com (Y.J.K.); cjstk2227@nate.com (M.K.); 4 Department of Pathology, Keimyung University School of Medicine, Daegu 42601, Korea; msc@dsmc.or.kr

**Keywords:** diabetic nephropathy, 3-hydroxybutyrate, oxidative stress, autophagy

## Abstract

Studies report beneficial effects of 3-hydroxybutyrate (3-OHB) on the treatment of type 2 diabetes and obesity, but the effects of 3-OHB on diabetic nephropathy have not been elucidated. This study was designed to investigate the efficacy and mechanism of 3-OHB against progression of diabetic nephropathy (DN). Mice (db/db) were fed normal chow, high-fat, or ketogenic diets (KD) containing precursors of 3-OHB. Hyperglycemia was determined based on random glucose level (≥250 mg/dL). Fasting blood glucose and body weights were measured once a week. Twenty four-hour urine albumin to creatinine ratio was determined 5 weeks after the differential diet. Energy expenditure was measured 9 weeks after the differential diet. Body weights were significantly lower in the KD group than those in other groups, but no significant differences in fasting blood glucose levels among three groups were observed. Urine albumin to creatinine ratio and serum blood urea nitrogen (BUN) to creatinine ratio in the KD group were significantly lower than in other groups. Histologic and quantitative analysis of mesangial area suggested that KD delayed the progression of DN phenotype in db/db mice. Metabolic cage analysis also revealed that KD increased energy expenditure in db/db mice. In vitro studies with proximal tubular cells revealed that 3-OHB stimulated autophagic flux. 3-OHB increased LC3 I to LC3 II ratio, phosphorylation of AMPK, beclin, p62 degradation, and NRF2 expression. Moreover, we found that 3-OHB attenuated high glucose-induced reactive oxygen species (ROS) levels in proximal tubular cells. In vivo study also confirmed increased LC3 and decreased ROS levels in the kidney of KD mice. In summary, this study shows in both in vivo and in vitro models that 3-OHB delays the progression of DN by augmenting autophagy and inhibiting oxidative stress.

## 1. Introduction

Diabetic mellitus (DM) is the most common cause of end-stage renal disease worldwide. As the age of the population is increasing, the prevalence of DM is rapidly increasing. Accordingly, the prevalence of diabetic nephropathy (DN) is also escalating [1]. DN is defined as increased albuminuria determined by albumin–creatinine ratio >30 ug/mg and/or glomerular filtration rate (GFR) <60 mL/min/1.73 m^2^ [2]. Korea National Health and Nutritional Examination Survey (KNHANES) reported that 3 in 10 patients over the age of 30 with DM present albuminuria or deceased renal function from the year 2013 to 2014 [3]. Various mechanisms have been suggested to explain the pathophysiology of DN [4]. Hyperglycemia-induced signaling pathways, such as the polyol pathway and hexosamine pathway, that lead to apoptotic processes in DN have been suggested. Hyperglycemia-stimulated production of advanced glycation end products and oxidative stress resulting in inflammation in susceptible cells have also been suggested [5,6]. 

The ketone body 3-hydroxybutyrate (3-OHB) is a major component of ketone bodies quantitatively and qualitatively [7]. Long considered as an alternative source during fasting or exercise, overwhelming evidence has accumulated that 3-OHB is not just an intermediate metabolite derived from fatty acid oxidation [8,9,10,11]. It functions as a signaling molecule that regulates various cellular metabolism with implications from obesity, diabetes mellitus and aging to cardiovascular diseases and cancer [12,13]. 

Ketogenic diet (KD) is a diet that primarily consists of high fats (55–60%), moderate proteins (30–35%), and very low carbohydrates (5–10%) [14]. KD was initially developed to treat pediatric patients with refractory epilepsy in 1921 and has been a very successful treatment option for intractable epilepsy [15]. Over recent years, however, beneficial effects of KD other than as a treatment option for intractable epilepsy have been firmly established. Studies show that KD is very effective in lowering body weights as well as maintaining blood glucose levels [16,17]. Given that obesity is a high-risk factor for the development of DN [18] and KD may reverse pathological processes of DN [19], we designed the current study to explore the possibility of KD being a potential therapeutic modality for the treatment of DN and to determine the mechanistic details of KD for the treatment of DN.

## 2. Materials and Methods

### 2.1. Animals

**Figure 1:**
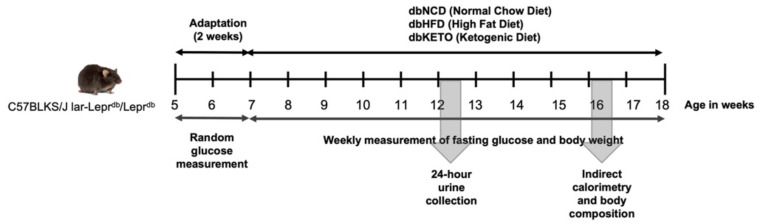
Graphical representation of the in vivo experiment.

To establish an animal DN model, male C57BLKS/J lar-Leprdb/Leprdb mice (5 weeks old, *n* = 27) were purchased from Jackson Laboratory (Bar Harbor, ME, USA). Mice were fed normal chow diet for 2 weeks for acclimation. When random glucose level exceeded 250 mg/dL, mice were randomly assigned into three groups, dbNCD, dbHFD, and dbKETO, according to the type of diet. dbNCD mice were fed normal chow diet, the composition of which, expressed as % of total kcal, is 55% carbohydrate, 25% protein, and 20% fat. dbHFD mice were fed a high-fat diet, the composition of which is 35% carbohydrate, 20% protein, and 45% fat. dbKETO mice were fed KD, the composition of which is 10% carbohydrate, 10% protein, and 80% fat. dbNCD and dbHFD were used as control groups since the aim of the current study was to determine the effect of 3-OHB on diabetic nephropathy. KD was synthesized according to the previous report [20]. In brief, 200 g of ground standard chow diet was mixed with 50 mL of (+)-1,3-butanediol (B84785, Sigma-Aldrich, St. Louis, MO, USA) and shaken to form round pellets. Normal chow and high-fat diets do not contain (±)-1,3-butanediol. Body weight and fasting glucose were measured once a week. Twenty-four-hour urine was obtained 7 weeks after differential diet feeding using a metabolic cage (Jeung Do Bio & Plant Co., Ltd., Seoul, Korea). Mice were sacrificed at 12 weeks after differential diet feeding. The overall experimental procedure is illustrated in Figure 1. This study was approved by the Institutional Animal Care and Use Committee (IACUC) of Keimyung University, School of Medicine, Daegu, Korea (KM-2017-41).

### 2.2. Indirect Calorimetry and Body Composition

Oxygen consumption (VO_2_), carbon dioxide production (VCO_2_), respiratory exchange ratios (RER), and energy expenditure were measured using an indirect calorimetry system PHENOMASTER (TSE System, Bad Homburg, Germany). Mice were maintained in each chamber at a constant environmental temperature of 22 °C. Lean body mass was assessed by 1H magnetic resonance spectroscopy (Bruker BioSpin) and energy expenditure was calculated per lean body mass (kcal/h/kg lean body mass).

### 2.3. Immunohistochemistry (IHC) Analyses

Tissue samples were fixed in paraformaldehyde, embedded in paraffin, and then cut into 4 μm thick sections. Sections were dried at 60 °C for 30 min and then deparaffinized with 3 washes of xylene for 5 min each. Sections were then rehydrated in graded alcohols, followed by incubation in 30% hydrogen peroxide for 30 min. Sections were incubated at 95 °C in 10 mM citrate buffer (pH 6.0) for antigen retrieval. Next, slides were incubated overnight at 4 °C with carboxymethyl lysine (CML, 1:50, Abcam, CA, USA), CD68 (Abcam, 1:50, CA, USA), LC3A/B (1:50, Cell Signaling Technology, Danvers, MA, UK) antibodies. Additionally, sections were incubated with secondary antibody (1:200, Santa Cruz Biotechnology, Santa Cruz, CA, USA) for 1 h at room temperature followed by staining with diaminobenzidine chromogen (Vector Laboratories, CA, USA) and counterstaining with hematoxylin. Stained sections were examined under light microscopy. Mesangial areas were quantified using a light microscope equipped with an imaging system (aMRc5, Carl Zeiss, Oberkochen, Germany).

### 2.4. Electron Microscopy (EM) Analyses

Kidney tissues were fixed in sodium cacodylate buffer for 2 h followed by washing three times and post-fixation with 1% osmium tetroxide for 2 h. Tissues were dehydrated in an ethanol and propylene oxide series, embedded and cut into ultrathin sections. Ultrathin sections were stained with uranyl acetated and lead citrate and viewed under electron microscope (H-7100; Hitachi, Tokyo, Japan). 

### 2.5. Blood Chemistry and Urine Analysis

Blood glucose levels were measured by tail vein prick and glucometer (Accu-Chek Active, Roche Diagnostics, Mannheim, Germany). Creatinine in urine was determined using the Quantichrome Urea Assay Kit (BioAssay System, Hayward, CA, USA). Urine albumin kit was measured using AssayPro (St. Charles, MO, USA).

### 2.6. Cell Culture

Human proximal tubular cell line HK-2 was cultured in RPMI 1640 or DMEM with low glucose concentration (5.5 mM) supplemented with 10% fetal bovine serum (FBS) and 1% antibiotic-antimycotic (Gibco, Grand Island, NY, USA). Cells were treated with 10 mM concentrations of sodium 3-hydroxybutyrate (3-OHB) (Sigma Aldrich, St. Louis, MO, USA). 

### 2.7. Real-Time Reverse-Transcriptase Polymerase Chain (RT-PCR) Analysis

Total RNA was extracted with Trizol reagent (Invitrogen, Carlsbad, CA, USA) according to the manufacturer’s protocol. Real-time RT-PCR analysis was performed using SYBR Green PCR Master Mix (TOYOBO, Osaka, Japan). The PCR reaction consisted of 10 μL of SYBR Green PCR Master Mix, 0.5 μL of 10 pmol/μL forward and reverse primers, 7 μL of water, and 2 μL of template cDNA in a total volume of 20 μL. Samples were assayed on a Light Cycler^®^ 96 (Roche, Germany) instrument.

### 2.8. Western Blot Analysis

Homogenized kidney tissues and lysed cell samples were boiled for 7 min with a gel-loading buffer (pH 6.8, 125 mM Tris-HCL, 4% sodium dodecyl sulfate (SDS), 10% 2-mercaptoethanol, and 0.2% bromophenol blue). Equal amounts of protein were separated by SDS–polyacrylamide gel electrophoresis (SDS-PAGE) using 8–5% gels. Gels were subsequently transferred onto a nitrocellulose membrane (GE Healthcare, Little Chalfont, UK). The membrane was placed in a blocking solution containing 5% skim milk with TBS-Tween (TBS-T) at room temperature and incubated with primary antibody overnight at 4 °C. Additionally, the membrane was washed for 30 min in TBS-T buffer and incubated with horseradish peroxidase-conjugated secondary antibodies (Santa Cruz Biotechnology, Santa Cruz, CA, USA) for 2 h at room temperature. Protein bands were detected by Super Signal West Pico Chemiluminescent Substrate (Thermo Fisher Scientific, Boston, MA, USA). Band densities were determined using LAS-3000 (Fujifilm, Tokyo, Japan).

### 2.9. Immunofluorescence

Cells were fixed with 4% paraformaldehyde for 20 min and incubated with PBS-T containing bovine serum albumin for 1 h at room temperature. Cells were incubated with NRF2 first antibody (1:200, Santa Cruz Biotechnology, sc-365949) overnight at 4 °C followed by incubation with fluorescence-labeled secondary antibody, Alexa Fluor 488 goat anti-mouse antibody (1:200, Invitrogen, A-11029). Cells were then counterstained with 4,6-diamidino-2-phenylindole (DAPI) and observed under a confocal microscope (LSM5, Carl Zeiss, Overkochen, Germany). Image J (https://imagej.nih.gov/ij, accessed on 24 January 2022) was used to quantify densities of figures.

### 2.10. Reactive Oxygen Species (ROS)

The amounts of ROS were determined by the formation of fluorescent 2,7-dichlorofluorescin diacetate (DCFDA). DCFDA was added to cells followed by incubation for 30 min. Cells were then suspended in the media after washing with PBS solution. ROS levels were observed under a fluorescence microscope (Axio observer A1, Carl Zeiss, Overkochen, Germany). Image J (https://imagej.nih.gov/ij, accessed on 24 January 2022) was used to quantify densities of figures.

### 2.11. Statistical Analysis

Experiments were repeated at least three times to gain consistent results. A one-way analysis of variance (ANOVA) was performed. Shapiro–Wilk statistics were used to verify the normal distribution, and Levene statistics were used to determine the homogeneity of variance. A Tukey test was undertaken for post hoc multiple comparisons. Student’s *t*-test was performed, and data were presented as mean ± standard deviation. In the non-normal distribution, a Kruskal–Wallis test was completed. The Statistical Package for the Social Science (SPSS) version 18.0 (SPSS Inc., Chicago, IL, USA) was used. The results were statistically significant at less than 0.05 of *p* value.

## 3. Results

### 3.1. KD Delays DN-Related Pathological Changes

**Figure 2:**
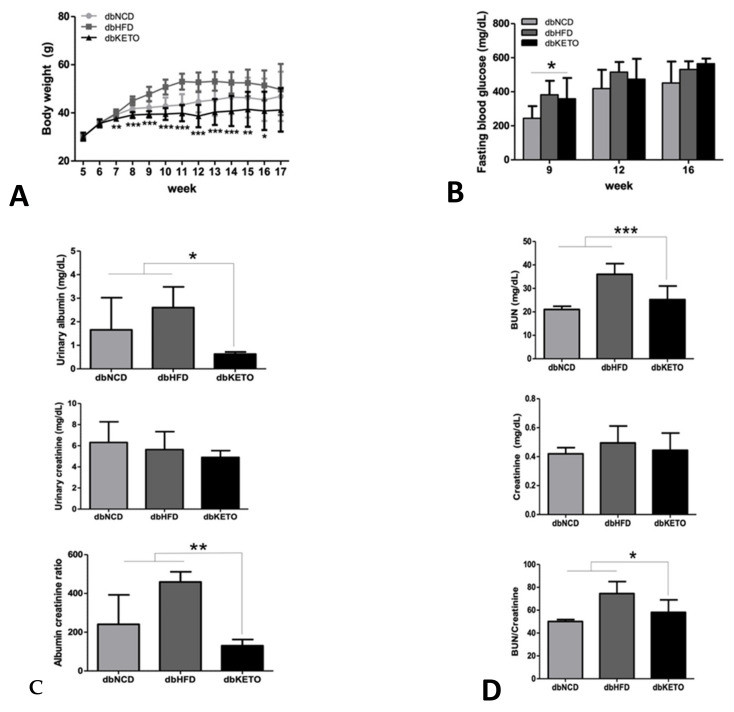

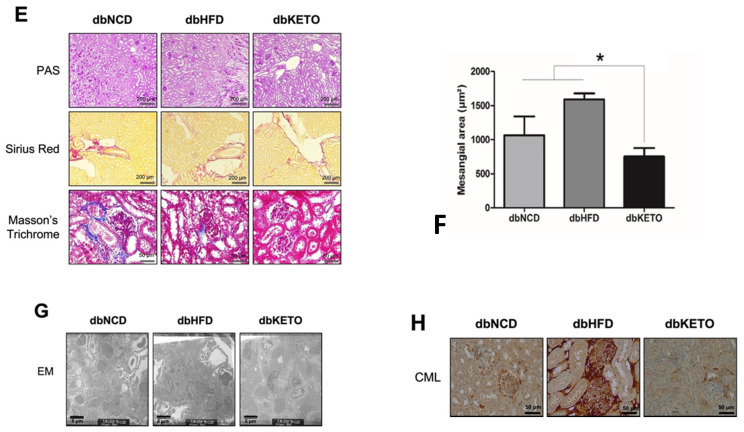
Biochemical and histological analyses. (**A**) Body weights of dbNCD, dbHFD, and dbKETO groups. (**B**) Fasting blood glucose (mg/dL) of dbNCD, dbHFD, and dbKETO groups. (**C**) Urinary albumin (top), urinary creatinine (middle), and albumin to creatinine ratio (bottom). (**D**) Blood urea nitrogen (BUN, top), creatinine (middle), and BUN to creatinine ratio (bottom). (**E**) Histologic analyses (PAS (top), Sirius red (middle), and Masson’s trichrome (bottom)) of the kidney of dbNCD, dbHFD, and dbKETO groups. (**F**) Quantification of mesangial area of dbNCD, dbHFD, and dbKETO. (**G**) Electron microscope (EM) analysis of kidney of dbNCD, dbHFD, and dbKETO. (**H**) Immunohistochemical analysis of carboxymethyl lysine (CML) of the kidney of dbNCD, dbHFD, and dbKETO groups. * *p* < 0.05, ** *p* < 0.01, *** *p* < 0.001.

Body weights in the dbKETO group were lower than in dbNCD and dbHFD groups throughout the entire experimental period (Figure 2A). Fasting blood glucose level was higher in the dbKETO group at 9 weeks. However, fasting glucose levels at 12 and 16 weeks were not different among three groups (Figure 2B). Notably, urinary albumin to creatinine and BUN to creatinine ratios, representative markers for renal functions, were lower in dbKETO than in dbNCD and dbHFD groups (Figure 2C,D). Immunohistochemical analyses (PAS, Sirius red, and trichrome) revealed decreased fibrotic changes in dbKETO compared with dbNCD and dbHFD groups (Figure 2E). Ultrastructural analysis using electron microscopy showed mild mesangial expansions in dbNCD and moderate mesangial expansion along with cellular proliferation in dbHFD. However, no pathological changes in mesangial structures were observed in dbKETO (Figure 2G). Quantification of mesangial area also supports ultrastructural analysis of mesangium, showing increased mesangial areas in dbNCD and dbHFD groups (Figure 2F). Histologic analysis of CML, a marker for advanced glycation end products, was also performed to determine the degree of the adverse effects of hyperglycemia (Figure 2H). Indeed, we observed that the level of CML in dbKETO was lower than in dbNCD and dbHFD.

### 3.2. KD Attenuates RER and Increases Energy Expenditure

**Figure 3:**
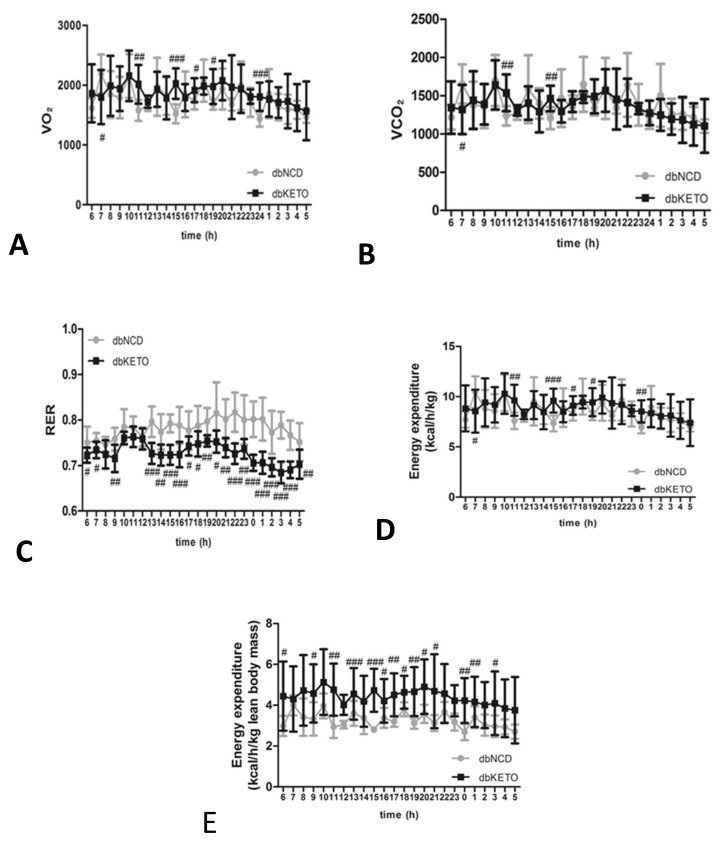
Metabolic properties of dbNCD and dbKETO groups. (**A**) O_2_ consumption. (**B**) CO_2_ production. (**C**) Respiratory exchange ratio (RER). (**D**) Energy expenditure per body weight (kg). (**E**) Energy expenditure per lean body mass. # *p* < 0.05, ## *p* < 0.01, ### *p* < 0.001.

To elucidate possible metabolic alterations in dbKETO, we conducted an additional experiment, the metabolic cage analysis, to determine the utilization of O_2_ and CO_2_ in the experimental groups (Figure 3). An RER value near 0.7 indicates that fat is predominantly used as a fuel source. Metabolic cage analysis revealed more fat-dominant oxidation in dbKETO than in dbNCD since the average RER value in dbKETO was 0.72 and in dbNCD 0.79. The dbKETO group consumed higher O_2_ and produced more CO_2_ than the dbNCD group (Figure 3A,B). Notably, we also found that energy expenditure per lean body mass in dbKETO group was higher than that in the dbNCD group (Figure 3E). 

### 3.3. KD Increases Autophagy in db/db Mice

**Figure 4:**
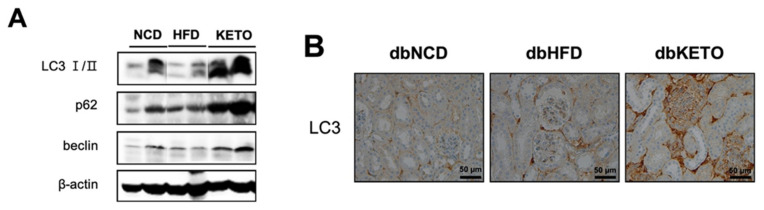
Expression of autophagy markers in the kidney of dbNCD, dbHFD, and dbKETO groups. (**A**) Representative figures showing expression levels of LC3 I/II, p62, and beclin in the kidney. (**B**) Immunohistochemical analysis of LC3 in the kidney of dbNCD, dbHFD, and dbKETO groups.

Increased energy expenditure in the dbKETO group prompted us to explore the possibility of 3-OHB to stimulate energy expenditure through regulating autophagy, the process of cellular self-eating. Given that the kidney has the second highest oxygen consumption after the heart and the proximal tubules require more energy than others since they reabsorb 80% of the filtrate that passes through the glomerulus [21] and the recently elucidated role of autophagy in energy metabolism [21,22], we hypothesized that 3-OHB stimulates autophagy in the kidney that might delay the pathologic progression of DM nephropathy. We tested our hypothesis first by determining molecules in the autophagic pathway in the kidney tissues. Additionally, we found that expression of LC3 (Map1lc3a), p62 (Sqstm1), and beclin (Atg6) was increased in the dbKETO group as compared to other experimental groups (Figure 4A). Consistently, IHC analysis of LC3 also revealed increased expression of LC3 in the dbKETO group (Figure 4B). 

### 3.4. 3-OHB Increases Autophagy in Renal Proximal Tubular Cells

**Figure 5:**
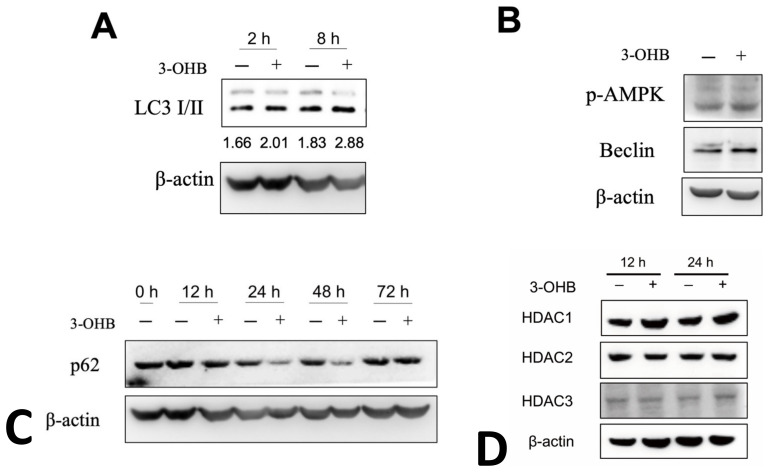
Expression of autophagy markers in HK-2 proximal tubular cells. (**A**) HK-2 cells were treated with/without 10 mM of 3-hydroxybutyrate (3-OHB) and incubated for the indicated times. Representative figures showing expression levels of LC3 I/II and LC3 I to II ratio as indicated as numbers below corresponding bands. (**B**) HK-2 cells were treated with/without 10 mM of 3-OHB and incubated for 24 h. Representative figures showing expression levels of phosphorylated AMP activated protein kinase (p-AMPK) and beclin. (**C**) HK-2 cells were treated with/without 10 mM of 3-OHB and incubated for indicated times. Representative figures showing expression levels of p62. (**D**) HK-2 cells were treated with/without 10 mM of 3-OHB and incubated for indicated times. Representative figures showing expression levels of histone deacetylase (HDAC) 1, 2, and 3.

To elucidate further the mechanistic details of the effect of KD on the kidney function, we performed an in vitro study using the human proximal tubule cell line HK-2 cells. 3-OHB treatment increased LC3I to LC3II ratios in non-serum starved HK-2 cells at 2 and 8 h (Figure 5A). Additionally, 3-OHB treatment increased phosphorylation level of AMP-activated protein kinase (AMPK, Prkaa) and beclin, markers of autophagy (Figure 5B). Furthermore, we observed that 3-OHB accelerates the degradation of p62 evidenced by the decreased expression of p62 after 12, 24, and 48 h of 3-OHB treatment (Figure 5C). With the previous studies that revealed that 3-OHB is an endogenous histone deacetylase (HDAC) inhibitor [9] and HDAC inhibitors activate autophagy in proximal tubular cells [23], we next determined the expression levels of HDAC1, 2, and 3 in 3-OHB treated HK-2 cells. Contrary to our expectation, we found that 3-OHB treatment did not change the expression levels of HDCA1, 2, and 3 (Figure 5D).

### 3.5. 3-OHB Increases NRF2 Expression in Renal Proximal Tubular Cells

**Figure 6:**
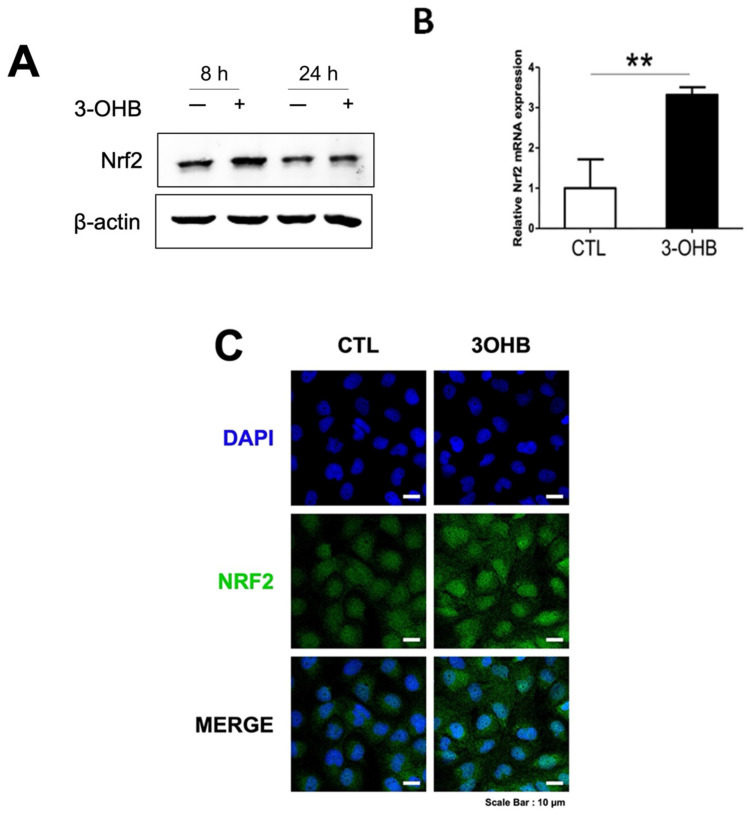
Effect of 3-hydroxybutyrate (3-OHB) on NRF2 expression in HK-2 cells. (**A**) HK-2 cells were treated with/without 10 mM of 3-OHB and incubated for the indicated times. A representative figure showing expression levels of NRF2. (**B**) HK-2 cells were treated with/without 10 mM of 3-OHB and incubated for 12 h. Cellular RNA was extracted and NRF2 expression level was determined by real time-RT PCR. (**C**) Fluorescence images of NRF2 (green). DAPI, 4,6-diamidino-2-phenylindole; ** *p* < 0.01.

Previous studies indicate that 3-OHB exerts an antioxidative response [9]. To further investigate the underlying mechanism of 3-OHB in stimulating autophagy, we determined the level of NRF2 (Nfe2l2), a transcription factor that activates expression of antioxidant enzymes and a molecule that is involved in both autophagy and antioxidant response [24]. Expectantly, we observed that 3-OHB increased expression levels of both NRF2 mRNA and protein (Figure 6A,B). Immunofluorescent analysis also indicated increased NRF2 expression in the nucleus of 3-OHB-treated HK-2 cells (Figure 6C).

### 3.6. KD and 3-OHB Decreases ROS Production

**Figure 7:**
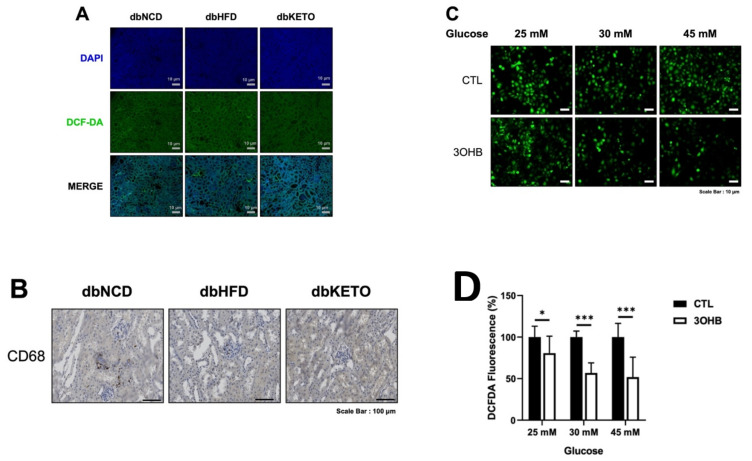
Ketogenic diet **(**KD) and 3-hydroxybutyrate (3-OHB) decreases ROS production. (**A**) Kidney tissues were stained with 2,7-dichlorofluorescin diacetate (DCFDA) and analyzed under fluorescence. (**B**) Kidney tissues were stained with CD68. (**C**) HK-2 cells were cultured in the high glucose (25, 30, and 45 mM) media for 24 h and then treated with 3-OHB for 24 h. ROS levels were determined with DCFDA and analyzed under a fluorescence microscope. (**D**) Quantification of fluorescence intensities of Figure 6C. DAPI, 4,6-diamidino-2-phenylindole * *p* < 0.05, *** *p* < 0.001.

ROS level in the kidney of dbKETO was lower than dbNCD and dbHFD groups (Figure 7A). Moreover, CD68, a marker for circulating macrophages, also decreased in the dbKETO group compared with dbNCD and dbHFD groups, implicating attenuated inflammatory processes in the dbKETO group (Figure 7B). We then examined the effect of 3-OHB on high glucose (25, 30, and 45 mM)-induced ROS production in HK-2 cells and found 3-OHB markedly reduced high glucose-induced ROS production (Figure 7C,D). 

## 4. Discussion

In the current study, we demonstrated in vitro that 3-OHB, a major ketone body quantitatively and qualitatively, stimulates autophagy and attenuates ROS production in renal proximal tubular cells. We also showed in vivo that KD ameliorates DN-related pathological change in db/db mice possibly via activation of autophagy and suppression of ROS production.

Treating patients with DN is challenging. DN is the leading cause of end-stage renal disease (ESRD) in most developed countries accounting for 50% of ESRD in the Republic of Korea [25]. The presence of DN doubles the risk for diabetes associated all-cause mortality as compared with the absence of DN [26]. Evidence indicates that intensive glycemic control in patients with DN poses a 41% increase in cardiovascular mortality and a 31% increase in all-cause mortality [26]. This might be due to 4–5 times higher incidence of severe hypoglycemia, a side effect of intensive glycemic control [27]. Randomized controlled studies also show that severe hypoglycemia predicts later mortality [28,29,30].

DN is characterized by persistent albuminuria and decreased glomerular filtration rate. Glomerulosclerosis, mesangial expansion, and tubulointerstitial fibrosis are the typical features of DN. In the current study, we showed that 3-OHB attenuates the pathological features of DN, fibrosis, and albuminuria as well as BUN. These results support those of a previous study that showed reversal of DN by KD by Poplawski et al. [19]. In the current study, the fasting glucose level in dbKETO remained unchanged and body weights decreased while in the study of Poplawski et al., the fasting glucose level decreased and body weight remained unchanged. These differences could be due to the usage of different precursors for 3-OHB. We used 1,3-butanediol as a precursor for 3-OHB while the study of Poplawski et al. used commercially available KD in which lard, butter, and corn oil were used. In support of our reasoning, evidence showed decreased body weight and increased energy expenditure in 1,3-butanediol-fed mice compared to control mice [10]. Other possibilities that could account for the difference in fasting glucose are plasma 3-OHB level and progression of DN [19]. Although we did not measure plasma 3-OHB level, studies showed 1,3-butanediol KD increases plasma 3-OHB level to 0.51–1 mM in C57BL/6-based mice strains [10,20], which is lower than plasma 3-OHB level (1.8~2.3 mM) in the study of Poplawski et al. Additionally, in the current study, we observed more profound histological findings of DN than those in the study of Poplawski et al., which could affect the regulatory role of 3-OHB on plasma glucose level.

Interesting but not unexpected is that energy expenditures per lean body mass in the dbKETO group are considerably higher than those in the dbNCD group. We reasoned that increased energy expenditure might be linked to activation of autophagy based on the results of a previous study that showed that increased energy expenditure impacts autophagy [31]. 

The pathophysiology of DN, how DM progresses into DN, is still elusive. Since glomerulus is the primary site of diabetic injury and glomerular hypertrophy and podocyte loss are hallmarks of DN, many studies have focused on the function of the glomerulus in relation to podocytes. Recently, however, studies that emphasize the tubular functions in regulating glomerular filtration have emerged [32,33,34]. This theory explains that DN may develop in relation to tubular dysfunction in regulating glomerular filtration [35]. Additionally, this alternative theory provides a convincing explanation for the therapeutic effects of sodium glucose cotransporter 2 (SGLT2) inhibitors, the only proven disease-modifying treatments for DN.

With this above-referenced evidence, we employed in the current study human proximal tubule cells, HK-2, to evaluate the potential therapeutic effect of 3-OHB in the treatment of DN. Maintaining basal level of autophagy is essential for renal homeostasis [36]. Additionally, impaired autophagy is observed in high glucose-treated proximal tubular cells, mice models of DN, and kidney tissue samples of human DN [37]. Dysregulated autophagy contributes to the progression of DN [38]. We showed in this study that 3-OHB augments the flow of autophagy in an in vitro model. We observed that the rate of degradation of p62 is faster than that of control in HK-2 cells. We also showed that 3-OHB stimulates autophagy in vivo. We found significantly increased levels of p62 and LC3 in the kidney of the dbKETO group. These results implicate the autophagy-mediated effect of 3-OHB on the delayed progression of DN. 

P62, also known as sequestosome 1 (SQSTM1), is a protein that binds ubiquitylated proteins and delivers them to autophagosomes. One of the proteins that is delivered to the autophagosome by p62 is Keap 1, an E3 ubiquitin ligase adaptor protein that guides NRF2 to ubiquitin-mediated proteosomal degradation [24]. Given that 3-OHB stimulates the expression of p62, we explored the possibility of increased p62 to sequester Keap 1 and leave NRF2 unubiquitylated. Indeed, we observed an increased level of NRF2 and consequently decreased production of ROS in 3-OHB-treated HK-2 cells.

3-OHB is an endogenous HDAC inhibitor and evidence indicated that 3-OHB exerts a protective effect against oxidative stress via selective depletion of HDAC1 and HDAC2 [9]. Thus, we determined the possible inhibitory effect of 3-OHB on the expression of HDAC and, to our surprise, found that the expression levels of HDAC1, HDAC2, HDAC3, and HDAC6 did not change. This result suggests that in renal proximal tubular cells, 3-OHB does not function as an endogenous HDAC inhibitor. However, since other HDACs (HDAC4, HDAC5, and HDACs7 through 11) were not examined in this study, the possibility that other HDACs might be inhibited by 3-OHB cannot be ruled out.

## 5. Conclusions

In summary, we showed the potential therapeutic usage of 3-OHB in the treatment of DN. KD attenuates albuminuria and BUN, and fibrosis, characteristic features of DN and 3-OHB stimulates autophagy and mitigates ROS production in renal proximal tubular cells. To the best of our knowledge, this is the first study to show a stimulatory effect of 3-OHB on autophagy in the kidney, an effect that might be linked to the protective effect of 3-OHB against the progression of DN. Further studies to evaluate the efficacy and underlying mechanistic details of 3-OHB for other renal diseases, including acute kidney injury and glomerulonephritis, will be of great merit for further scientific understanding and clinical translation. 

## Data Availability

Data is contained within the article.
